# Comparative Effectiveness of Different Anticoagulation Strategies in Atrial Fibrillation and Renal Dysfunction: A Systematic Review

**DOI:** 10.7759/cureus.48072

**Published:** 2023-10-31

**Authors:** Kunj R Ghantiwala, Archi Dhamelia, Dhwani S Vaghani, Binay K Panjiyar

**Affiliations:** 1 Medicine, Government Medical College, Bhavnagar, Bhavnagar, IND; 2 Internal Medicine, Mahatma Gandhi Mission (MGM) Medical College, Navi Mumbai, Navi Mumbai, IND; 3 Internal Medicine, Mahatma Gandhi Mission (MGM) Medical College and Hospital, Aurangabad, Aurangabad, IND; 4 Global Clinical Scholars Research Training (GCSRT) and Postgraduate Medical Education (PGMEE), Harvard Medical School, Boston, USA; 5 Internal Medicine, California Institute of Behavioral Neurosciences & Psychology, Fairfield, USA

**Keywords:** end-stage renal disease (esrd), chronic kidney disease (ckd), renal dysfunction, atrial fibrillation (af), anticoagulation strategies

## Abstract

Atrial fibrillation (AF) is a common cardiac arrhythmia that increases the risk of stroke and thromboembolism. Anticoagulation therapy can reduce this risk, but the optimal choice of anticoagulant in patients with AF and renal dysfunction is challenging. Renal dysfunction is a common comorbidity seen in patients with AF. Renal dysfunction would affect the pharmacokinetics and pharmacodynamics of anticoagulants and make the patient more prone to bleeding complications. This complicates the assessment of the risks, benefits, and ratio for starting anticoagulant drugs in patients with renal dysfunction. Therefore, there is always a therapeutic conundrum due to the increased risk of bleeding and thromboembolic events in AF patients with renal dysfunction.

We conducted a systematic review to summarize the current literature and identify the challenges of anticoagulation strategies in AF with renal dysfunction. We examined 180 articles from reputable journals published from 2018 to June 2023 and selected eight papers for detailed analysis.

The studies we chose included a variety of drug treatments, such as traditional therapies like vitamin K antagonists, factor Xa inhibitors, heparins, and direct thrombin inhibitors. This systematic review will provide comprehensive information on the latest data on the effectiveness of various pharmacological treatments (anticoagulation strategies) in AF patients with renal dysfunction. The aim is to help doctors and other healthcare decision-makers choose the best anticoagulation strategy in AF patients with renal dysfunction and to overcome their dilemma between bleeding risk and systemic thromboembolic events.

## Introduction and background

Atrial fibrillation (AF) is a common arrhythmia that increases the risk of stroke and systemic embolism. Compared to those with normal or near-normal kidney function, patients with renal dysfunction or chronic kidney disease (CKD) are at very high risk for cardiovascular morbidity and mortality, including AF. AF is less common in the general population, with less than 2% prevalence. However, those with CKD have a much higher prevalence of AF, with more than 10 times the occurrence rate. Patients with moderate (stage 3) CKD have almost 20% of AF prevalence. For individuals with more advanced CKD (stage 4 or 5), especially those requiring dialysis, the incidence of AF is even higher, with some studies indicating that nearly 33% of all patients have AF [1]. According to society guidelines, anticoagulation therapy is needed for patients with AF and high stroke risk factors, like a previous stroke, a transient ischemic attack (TIA), or a CHA2DS2-VASc. However, there is still confusion regarding the selection of anticoagulation in CKD patients [2].

Having CKD increases the possibility of developing health conditions that usually need anticoagulation such as venous thromboembolic events and AF. However, it also significantly raises the likelihood of experiencing complications related to anticoagulation, particularly major bleeding [3]. Assessing the risk/benefit ratio of anticoagulation for persons with renal dysfunction, especially those with more advanced renal dysfunction and AF, is complicated. There is limited evidence of clinical benefit from anticoagulant drugs, but there is a clear indication of increased risk of death and bleeding problems [4].

There are conflicting reports on the effectiveness of oral anticoagulants in lowering the risk of stroke in patients with advanced renal dysfunction and AF. While a large study in Denmark found a reduced risk of ischemic stroke, another analysis of patients treated with warfarin showed a two-fold increase in stroke risk [5].

For the treatment of venous thromboembolism (VTE) and AF in CKD patients, vitamin K antagonists (VKA) have traditionally been used. However, VKAs have certain drawbacks in this population, such as difficulty maintaining therapeutic levels, regular monitoring needs, potential risks of vascular calcification, and the rare condition of calciphylaxis [5]. Direct oral anticoagulants (DOACs) are becoming more popular for treating both VTE and AF, but they have not been studied in randomized controlled trials (RCTs) for severe renal impairment. Low-molecular-weight heparins (LMWHs) have been commonly used to initially treat VTE and are the primary therapy for VTE prophylaxis, but they have not been well studied in individuals with severe renal impairment [5].

This systematic review will help with anticoagulation on the available evidence of the effectiveness and risks of anticoagulation strategies in AF patients with renal dysfunction.

## Review

Methods

**Figure 1:**
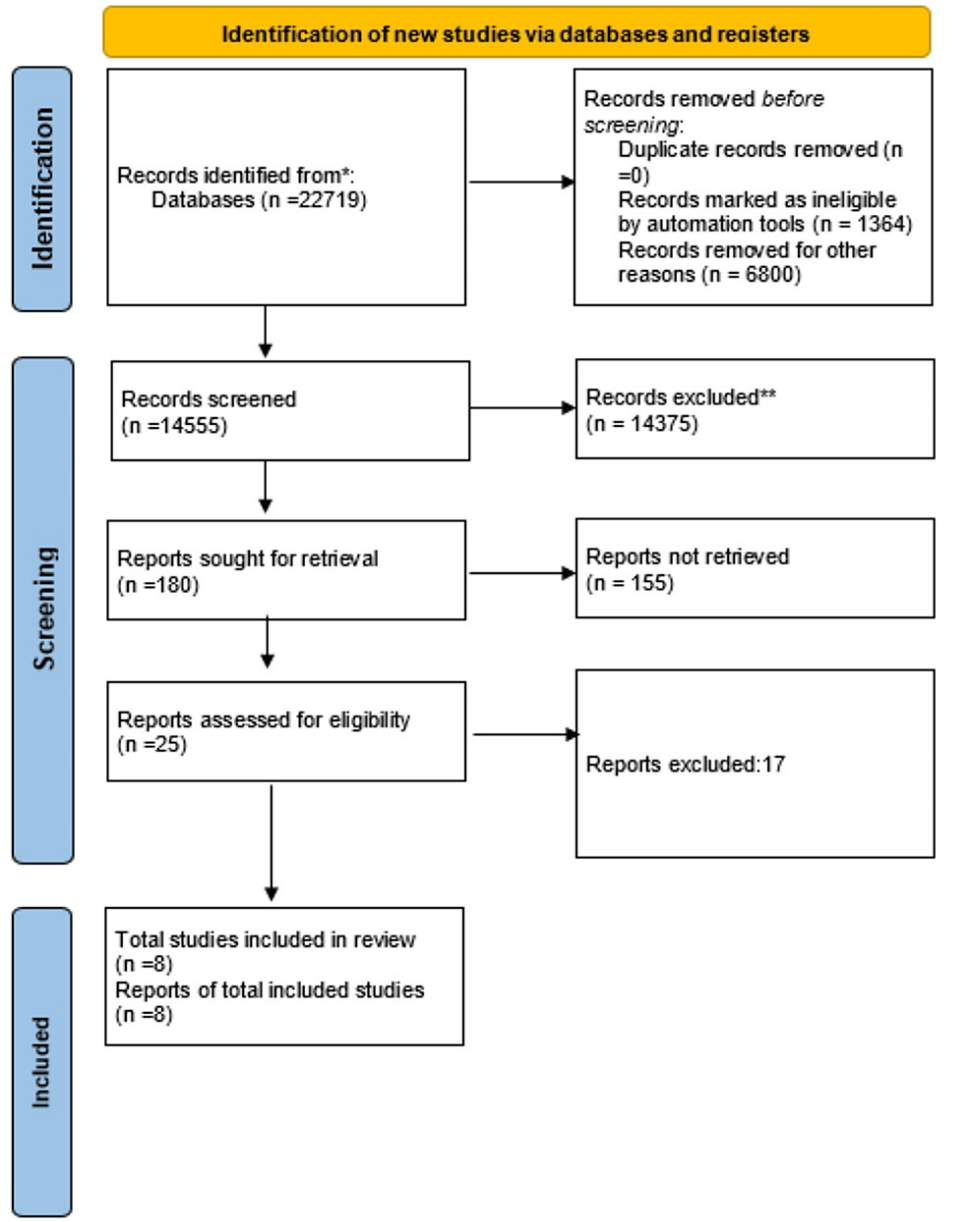
PRISMA flow diagram illustrating the search strategy and study selection process for the systematic review PRISMA: Preferred Reporting Items for Systematic Reviews and Meta-analyses.

This review focuses on clinical research involving the administration of anticoagulants to patients with AF and renal disease. Animal studies and articles that presented no clinical data but rather discussed the methods of anticoagulation regimens in AF with renal failure were omitted. The review adheres to the standards outlined in Figure 1 for Preferred Reporting Items for Systematic Reviews and Meta-analyses (PRISMA) [6] for 2020 and solely makes use of information gathered from articles that have already been published, obviating the requirement for ethical review.

Systematic Literature Search and Study Selection

We used PubMed, MEDLINE, and Google Scholar to search for pertinent papers thoroughly. On PubMed, we looked for research that was cited in reviews, editorials, and commentaries. But even so, we kept looking for further papers that met our inclusion standards.

We independently evaluated a list of abstracts for inclusion according to predetermined standards. The requirements included using anticoagulant techniques, concentrating on AF with renal failure, and including a clinical cohort in the study that was precisely characterized. Review articles and animal research were not included. A dual review was performed by six reviewers, and any disputes were settled through conversation.

Inclusion and Exclusion Criteria

In order to accomplish our research objectives, we defined precise inclusion and exclusion criteria. Table 1 provides an overview of our criteria.

**Table 1:** The criteria adopted during the literature search process

Inclusion criteria	Exclusion criteria
Human studies	Animal studies
Adults, age: 19+ years	Children, age < 19 years
Last 5 years	Before 2018
Gender: all	
Free papers	Paid articles
Language: English	Other languages

Search Techniques

A complete literature review was conducted using the population, intervention/condition, control/comparison, and outcome (PICO) criteria. Acute anticoagulation strategies, AF, and renal dysfunction were used as relevant keywords in the search on databases like PubMed (including MEDLINE) and Google Scholar libraries. A thorough search strategy was created using the medical subject heading (MeSH) approach for PubMed (including MEDLINE) and Google Scholar, as shown in Table 2.

**Table 2:** The search strategy, search engines used, and the number of results displayed

Database	Search strategy	Search result
PubMed	Anticoagulation strategies or atrial fibrillation or renal dysfunction	5819
Google Scholar	Anticoagulation strategies or atrial fibrillation or renal dysfunction	16900

Quality Appraisal

We made use of a variety of quality assessment tools to ensure the validity of the papers we chose. For systematic reviews and meta-analyses, we used the PRISMA checklist and Cochrane bias tool assessment for randomized clinical trials. The Newcastle-Ottawa tool scale was used to evaluate clinical studies that were not randomized. Using the Critical Appraisal Skills Program (CASP) checklist, we evaluated the caliber of the qualitative investigations, as shown in Table 3. We used the Scale for the Assessment of Narrative Review Articles (SANRA) to rate the article's quality in order to prevent any ambiguity in the classification.

**Table 3:** Quality appraisal tools used PRISMA: Preferred Reporting Items for Systematic Reviews and Meta-analyses; SANRA: Scale for the Assessment of Non-systematic Review Articles.

Quality appraisal tool	Type of studies
Cochrane Bias Tool assessment	Randomized control trials
Newcastle-Ottawa Tool	Non-RCT and observational studies
PRISMA checklist	Systematic reviews
SANRA checklist	Any other without a clear method section

Results

After selecting databases such as PubMed, MEDLINE, and Google Scholar, we retrieved 22719 articles in July 2023. These papers were then carefully scrutinized, applying specific criteria to exclude 8164 articles. Out of the remaining 14555 papers, 14375 were eliminated due to inadequate titles or abstracts. We evaluated the remaining 180 papers and disqualified 172 articles as they did not meet our inclusion criteria. Finally, we conducted a thorough quality check on the remaining eight papers, which all fulfilled our criteria. Our systematic review includes these eight articles, and Table 4 provides comprehensive descriptions of each paper.

**Table 4:** Summary of the results of the selected papers ESRD: End-stage renal disease; DOAC: Direct-acting oral anticoagulants; OAC: Oral anticoagulants; AF: Atrial fibrillation; CKD: Chronic kidney disease; ESKD: End-stage kidney disease; CrCl: Creatinine clearance.

Author/year	Country	Study design	Database used	Conclusion
Stanifer et al./2020 [1]	USA	Randomized controlled trial	PubMed	Apixaban was found to cause less bleeding than warfarin if the CrCl is 25-30 mL/min in the AF patient.
Bhatia et al./2018 [2]	USA	Review article	PubMed	Use DOAC over warfarin in AF with CKD.
Pokorney et al./2020 [5]	USA	Retrospective cohort	Google Scholar	In patients with ESRD and AF, OAC use was not associated with a reduction in the risk of ischemic stroke.
Yao et al./2020 [7]	USA	Retrospective cohort	PubMed	Apixaban, dabigatran, and rivaroxaban were associated with a lower risk of stroke, major bleeding, and mortality than warfarin.
Cheung and Wong/2020 [8]	USA	Editorial commentary on the results of a meta-analysis	PubMed	Use DOAC over warfarin in AF with renal impairment.
Kumar et al./2019 [9]	USA	Review article	Google Scholar	Oral anticoagulation in CKD patients is contentious due to their increased propensity to both thrombosis and bleeding.
Hu et al./2018 [10]	USA	Review article	PubMed	Proper dosing, efficacy, and safety of anticoagulants in patients with advanced renal dysfunction are sparse and almost exclusively observational.
Belley-Cote and Eikelboom/2020 [11]	Canada	Invited commentary on the results of a meta-analysis	Google Scholar	Use a left atrial appendage occlusion device in patients with atrial fibrillation and ESRD.

Discussion

As far as we are aware, this study has been done thus far comparing several oral anticoagulants throughout the spectrum of kidney function. The study discovered that as patients' renal function deteriorates, direct oral anticoagulants (DOACs) are taken progressively less frequently than warfarin. To assist in clinical decision-making, there are no firm clinical guidelines generated from RCTs, and the results of observational research are inconsistent [9].

Pathophysiology of the Thromboembolic Event in AF Patients With Renal Dysfunction

The increased risk of thromboembolism in individuals with concurrent CKD and AF is a result of changes to the three elements of Virchow's triad. A hypercoagulable condition is brought on by altered prothrombotic blood elements and elevated platelet activity. Deranged hemodynamics are caused by changes in arterial wall contractility as a result of inflammation, changes in atrial architecture and contractility, and activation of the renin-angiotensin-aldosterone system (RAAS). The production of thrombi is predisposed by damage to the dysfunction of the heart muscle and vascular endothelium. The separate effectors and the elements of the triad interact significantly [9].

There are kidney tests like serum creatine test, cystatin c, glomerular filtration rate (GFR), blood urea nitrogen (BUN), kidney ultrasound, kidney biopsy, etc. The best overall indicator of the glomerular function is the GFR. The glomerular filtration rate shows how well the kidneys are filtering; the lower value means the kidneys are not working properly. The staging of CKD based on the GFR is as follows [12] - Stage 1: kidney damage with normal kidney function with 90 or higher GFR; Stage 2: Kidney damage with mild loss of kidney function with 89-60 GFR; Stage 3: mild to severe loss of kidney function with 59-30 GFR; Stage 4: severe loss of kidney function with 29-15 GFR; Stage 5: kidney failure with less than 15 GFR.

Classification of Anticoagulants

**Figure 2:**
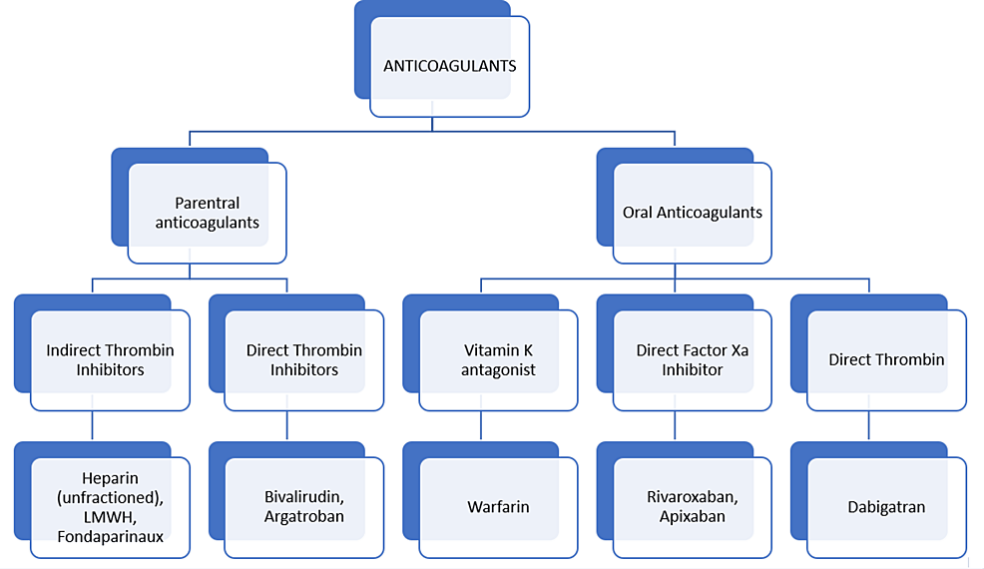
Classification of anticoagulant drugs LMWH: Low-molecular-weight heparin. Source: This image of classification was created by Kunj R. Ghantiwala, the author of this paper.

Figure 2 shows the classification of anticoagulant drugs.

Mechanism of Action of Anticoagulants

Unfractionated heparin (UFH): UFH is a mixture of polysaccharide chains that enhance the activity of antithrombin, a natural inhibitor of coagulation that blocks thrombin, and FXa, two key factors in the coagulation cascade. UFH binds to antithrombin through a pentasaccharide sequence and increases its anticoagulant effect [13].

LMWH: LMWHs are anticoagulant drugs that are derived from UFH by chemical splitting. LMWHs have a similar mechanism of action as UFH but with fewer side effects and more predictable responses. LMWHs can be injected subcutaneously without constant monitoring and allow for outpatient treatment of some VTE patients.

VKA: VKAs block the enzyme that recycles vitamin K, which is needed for several coagulation factors to become active. VKAs can be taken orally, and warfarin is the most commonly used VKA for preventing thromboembolic events.

Fondaparinux, argatroban, and bivalirudin: Fondaparinux, argatroban, and bivalirudin are anticoagulant drugs that are designed to target specific clotting factors or enzymes. Fondaparinux is based on the active component of heparins and inhibits FXa; argatroban is derived from L-arginine and blocks thrombin; and bivalirudin is based on hirudin from leeches and also inhibits thrombin.

Direct oral anticoagulants: Direct oral anticoagulants (DOACs) are anticoagulant drugs that are taken orally and act quickly by blocking FXa or thrombin. DOACs have replaced VKAs in the clinic as they do not need the heparin bridging period, have a wider therapeutic window, and have fewer drug interactions. Ximelagatran was the first DOAC that was designed by computer modeling, but it was withdrawn from the market due to liver toxicity. Dabigatran is a direct thrombin inhibitor, which is derived from a compound that inhibits serine proteases, and it is used as an oral prodrug. FXa DOACs are anticoagulant drugs that target FXa, which was identified as a promising target in the 1980s. Some FXa DOACs are derived from natural compounds from leeches.

Side Effects of Anticoagulant Drugs

The side effects of the anticoagulant drugs are detailed below:

Warfarin: Warfarin has several adverse effects that are not related to bleeding, such as interference with vitamin K-dependent enzymes, acceleration of vascular calcification, heightened risk of calciphylaxis, and warfarin-related nephropathy [14]. Warfarin inhibits the activation of matrix G1a protein, which is a natural inhibitor of vascular calcification. This may explain why some studies have found an increased risk of ischemic stroke with warfarin. Warfarin has an impact on residual renal function. It can cause microvascular bleeding in the kidneys, leading to tubular obstruction and renal injury. This may affect the residual renal function of patients on dialysis, which may have a survival benefit.

Dabigatran: Dabigatran is a direct thrombin inhibitor that is mainly eliminated by the kidneys and has low protein binding. It has a higher risk of bleeding and lower efficacy in patients with end-stage kidney disease (ESKD) compared with warfarin. Dabigatran can be removed by dialysis, which may increase the risk of thromboembolic events or bleeding depending on the patient’s adherence to dialysis sessions. It may also cause kidney injury by inducing microvascular bleeding and tubular obstruction, similar to warfarin-related nephropathy.

Rivaroxaban: Patients bleeding from rivaroxaban also did not have an antagonist drug available, which may explain why there was an even higher observed risk for bleeding complications, which may lead to death in severe renal dysfunction patients as compared with warfarin. Andexanet alfa, a reversal agent for factor Xa inhibitors, was approved by the FDA in 2018.

Apixaban: It is the least reliant on kidney function for clearance and is minimally affected by dialysis. Apixaban has shown safety and efficacy in patients with advanced renal dysfunction down to a CrCl of 25 mL/min but not in ESKD patients. Apixaban has a lower risk of bleeding and stroke than warfarin and aspirin in patients with CKD but not in ESKD patients. Apixaban has a higher plasma concentration in ESKD patients than in healthy controls but is well tolerated. The FDA approved a dose reduction for ESKD patients.

Edoxaban: Edoxaban is eliminated by the kidneys and the liver, and its dose should be reduced in patients with moderate to severe kidney impairment. It is not approved for patients with ESKD or CrCl greater than 95 mL/min as it may cause higher bleeding or stroke risk. It is non-inferior to warfarin in preventing stroke and systemic embolism and superior in reducing bleeding and cardiovascular mortality.

Various anticoagulation strategies are being used to prevent thromboembolic events in AF patients with renal dysfunctions mostly VKAs and direct-acting oral anticoagulants.

In Individuals With CrCl of 15-30 mL/min

According to the AF guideline update, decreased dosages of apixaban, dabigatran, rivaroxaban, or edoxaban may be an option for people with CrCl of 15-30 mL/min [15]. Although there are limited efficacy and safety outcome data, both the US Food and Drug Administration and European Medicines Agency have approved reduced doses of direct factors Xa inhibitors like apixaban, edoxaban, and rivaroxaban in patients with severe renal dysfunction with an eGFR of 15-30 ml/min [9]. According to the American Heart Association (AHA), American College of Cardiology (ACC), and Heart Rhythm Society (HRS), warfarin is recommended, but we can also consider other DOACs. According to the Canadian Cardiovascular Society (CCS), warfarin is not recommended [2]. However, FDA-approved direct oral anticoagulant drugs and doses across the spectrum of kidney disease for the prevention of thromboembolic events in AF based on CrCl suggest 75 mg of dabigatran twice daily [10]. When given apixaban instead of warfarin, patients with CrCl of 25-30 mL/min experienced fewer major bleeding incidents and fewer major and clinically relevant non-major bleeding incidents, and patients on edoxaban (versus warfarin) whose CrCl fell below 30 mL/min after baseline had similar rates of stroke (thromboembolic events) and bleeding complications [1].

In Individuals With End-Stage Renal Disease and Dialysis (CrCl < 15 mL/min)

Dialysis-dependent patients with severe renal dysfunction had a higher risk of stroke than the normal functioning kidney population, but the increased risk of intracerebral hemorrhage is much higher than the increased risk of ischemic stroke [1]. According to AHA, ACC, and HRS, warfarin is recommended against other DOACs like dabigatran and rivaroxaban, but CCS says no anticoagulation therapy in dialysis patients [2]. Additionally, patients with severe renal dysfunction have higher rates of anemia, accelerated calcific atherosclerosis, uremic platelet dysfunction, and other uremic sequelae. These factors could all have a varied impact on these patients' risk of hemorrhagic or ischemic stroke [1]. You can also use a left atrial appendage occlusion device to avoid the side effects, monitoring the kidney function and dose of anticoagulants [11].

In Individuals With CrCl of More Than 30 mL/min

DOACs are generally safer and more effective than warfarin, especially when it comes to serious bleeding. DOACs cause half as much life-threatening bleeding as warfarin, and they are also more convenient than warfarin because they do not require frequent blood monitoring and can be given safely in fixed doses [16]. In the CrCl > 95, we can use 150 mg of dabigatran twice daily, 20 mg of rivaroxaban per day, or 5 mg of apixaban twice a day, but if the 50 < CrCl > 90, we should give 2.5 mg of apixaban twice daily or 60 mg of edoxaban per day [10]. In patients with more advanced renal impairment receiving hemodialysis, apixaban (5 mg BID) or 2.5 mg BID causes similar rates of bleeding events and strokes as warfarin as well as non-major bleeding events and mortality are higher in the use of apixaban than the use of warfarin [17].

Exposure to VKA damages kidney function in patients with AF and advanced renal dysfunction, leading to a decline in eGFR values [18]. In addition to the increased risk of bleeding complications associated with anticoagulation in patients with advanced renal dysfunction, using warfarin also gives additional risks related to the inhibition of vitamin K-dependent pathways, including worsening kidney function and pathways affecting dystrophic calcification. Patients with end-stage renal disease who need dialysis only include dose instructions for the new oral anticoagulants (NOACs) - rivaroxaban and apixaban [1].

Substitutes for Anticoagulation

For patients with AF and advanced renal dysfunction who cannot tolerate anticoagulation, one very effective anticoagulation substitute is left atrial appendage closure [19]. Recent research on patients with AF found that left atrial appendage closure was equally safe and effective in those with and without chronic kidney disease [20]. We can also use them in patients with AF without ESRD who have a contraindication to long-term anticoagulation; these devices seem to be non-inferior to anticoagulation [21]. Utilizing aspirin or aspirin and clopidogrel together is another viable approach; however, antiplatelet medication is not particularly beneficial in preventing strokes in people with AF and advanced renal dysfunction [11].

Limitations

We have constraints with our literature review. We especially restricted our research to English articles published during the last five years. Our research was restricted to English-language papers on anticoagulation, AF, and renal dysfunction, and we only used free articles. For more particular information, more study is required.

## Conclusions

Renal dysfunction and AF often coexist; this systematic review highlights the appropriate anticoagulation strategies in AF patients with renal dysfunction. In patients with coexisting AF and renal dysfunction, the anticoagulation strategy should be based on the patient's renal function or CKD stage. With decreased major bleeding and comparable efficacy, DOACs may be preferable to warfarin in patients with VTE or AF and a CrCl ≥ 30 ml/min. In those patients (CrCl < 15 ml/min), there are not enough pieces of evidence that suggest what to use in this situation. However, we should avoid using warfarin in these patients and use other anticoagulants. We can also use the left atrial appendage to prevent them from bleeding-side effects of anticoagulant drugs. Future randomized clinical trials are required to give more conclusive data, particularly in individuals with severely impaired kidney function, on the comparative efficacy and safety of various oral anticoagulant medications.
